# Involvement of kindlin‐2 in irisin’s protection against ischaemia reperfusion‐induced liver injury in high‐fat diet‐fed mice

**DOI:** 10.1111/jcmm.15910

**Published:** 2020-09-20

**Authors:** Jia Zhang, Yifan Ren, Jianbin Bi, Mengzhou Wang, Lin Zhang, Tao Wang, Shasha Wei, Xingyi Mou, Yi Lv, Rongqian Wu

**Affiliations:** ^1^ National Local Joint Engineering Research Center for Precision Surgery & Regenerative Medicine Shaanxi Provincial Center for Regenerative Medicine and Surgical Engineering First Affiliated Hospital of Xi’an Jiaotong University Xi’an China; ^2^ Department of Hepatobiliary Surgery First Affiliated Hospital of Xi’an Jiaotong University Xi’an China; ^3^ Zonglian College Xi’an Jiaotong University Health Science Center Xi’an China

**Keywords:** hepatic I/R, high‐fat diet, irisin, kindlin‐2, steatotic liver

## Abstract

Liver steatosis is associated with increased ischaemia reperfusion (I/R) injury. Our previous studies have shown that irisin, an exercise‐induced hormone, mitigates I/R injury via binding to αVβ5 integrin. However, the effect of irisin on I/R injury in steatotic liver remains unknown. Kindlin‐2 directly interacts with β integrin. We therefore suggest that irisin protects against I/R injury in steatotic liver via a kindlin‐2 dependent mechanism. To study this, hepatic steatosis was induced in male adult mice by feeding them with a 60% high‐fat diet (HFD). At 12 weeks after HFD feeding, the mice were subjected to liver ischaemia by occluding partial (70%) hepatic arterial/portal venous blood for 60 minutes, which was followed by 24 hours reperfusion. Our results showed HFD exaggerated I/R‐induced liver injury. Irisin (250 μg/kg) administration at the beginning of reperfusion attenuated liver injury, improved mitochondrial function, and reduced oxidative and endoplasmic reticulum stress in HFD‐fed mice. However, kindlin‐2 inhibition by RNAi eliminated irisin's direct effects on cultured hepatocytes. In conclusion, irisin attenuates I/R injury in steatotic liver via a kindlin‐2 dependent mechanism.

## 1 INTRODUCTION

The incidence of non‐alcoholic fatty liver disease (NAFLD) is increasing rapidly worldwide.^1^, ^2^, ^3^ In China, the national prevalence of NAFLD is estimated at 29.2% and the burden of NAFLD is expected to increase dramatically.^4^ Hepatic ischaemia reperfusion (I/R) injury is an inevitable complication associated with liver transplantation, partial hepatectomy and hypovolemic shock.^5^ Steatotic liver appears to be more sensitive to I/R injury.^6^ Fat‐laden hepatocytes are damaged by chronic oxidative/nitrosative stress, which is further increased during I/R, leading to extensive parenchymal damage.^7^

Irisin, an exercise‐induced and muscle‐secreted myokine, was first discovered for driving browning of white fat and thermogenesis in 2012.^8^ As then, many studies have revealed that irisin is also a potent antioxidant.^9^, ^10^, ^11^, ^12^, ^13^, ^14^, ^15^, ^16^ Our previous studies have also shown irisin improves mitochondrial function and decreases oxidative stress via binding to αVβ5 integrin in I/R injury.^17^, ^18^ However, the effect of irisin on I/R injury in steatotic liver remains unknown.

Kindlin‐2 is a focal adhesion protein that regulates integrin signalling and cell‐matrix adhesion.^19^ It directly interacts with the cytoplasmic tail of β integrin,^20^ which is recognized as a part of the irisin receptor.^21^ A recent study has shown kindlin‐2 expression is up‐regulated in human and mouse fibrotic livers and depletion of kindlin‐2 reduces CCL4‐induced liver injury in mice. However, the role of kindlin‐2 in irisin's biological function is currently unclear.

We therefore suggested that irisin attenuates hepatic I/R injury via a kindlin‐2 dependent mechanism in steatotic liver. The aim of the present study was to explore the effects and likely mechanisms of irisin on hepatic I/R injury in high‐fat diet (HFD)‐fed mice.

## 2 MATERIALS AND METHODS

### 2.1 Experimental animals and diets

Male wild‐type C57BL/6J mice (18 ± 3 g; 6‐8 weeks) were purchased from the Experimental Animal Center of Xi'an Jiaotong University and bred in a pathogen‐free environment under 12‐hour light‐dark cycle at a temperature of 23‐25°C. Standard chaw (Control diet; CD) or 60% high‐fat diet (D12492, Research Diets Inc) were provided for 12 weeks. All mice were treated according to the guidelines of the China Council on Animal Care and Use. This project was approved by the Institutional Animal Care and Use Committee of the Ethics Committee of Xi'an Jiaotong University Health Science Center, China.

### 2.2 Mouse model of hepatic I/R and experimental design

After 12 weeks on a control diet or high‐fat diet, the mouse hepatic I/R model was established as we described before.^17^ Briefly, mice were anaesthetized with isoflurane inhalation and maintained at a concentration of 1.5%‐2%. Liver ischaemia was induced by occluding partial (70%) hepatic arterial/portal venous blood for 60 minutes by a microvascular clip. Then, clip was removed and reperfusion began. Sham operation underwent all the procedures except hepatic ischaemia. There were five groups involved in present study: (a) CD‐Sham: CD‐fed mice underwent sham operation, and 0.5 mL saline was administrated intraperitoneally; (b) CD‐I/R: CD‐fed mice underwent hepatic I/R, and 0.5 mL saline was administrated intraperitoneally immediately after the initiation of reperfusion; (c) HFD‐Sham: also showed as sham group, HFD‐fed mice underwent sham operation and 0.5 mL saline was administrated intraperitoneally; (d) HFD‐I/R: also showed as vehicle group, HFD‐fed mice underwent hepatic I/R, and 0.5 mL saline was administrated intraperitoneally immediately after the initiation of reperfusion; (e) HFD‐irisin: also showed as irisin group, HFD‐fed mice underwent hepatic I/R, and irisin (250 μg/kg, 0.5 mL; 067‐29A, Phoenix Pharmaceuticals, Inc)^17^ was administrated intraperitoneally immediately after the initiation of reperfusion.

### 2.3 Haematoxylin and eosin (H&E) staining and oil red O staining

The liver sections fixed in 4% paraformaldehyde were embedded in paraffin. Then cut the paraffin blocks into 5 mm‐slices and stained with haematoxylin and Eosin. Liver injury score was evaluated as we described before.^17^ The frozen liver sections were stained with Oil Red O to evaluate hepatic fat content.

### 2.4 Measurement of serum alanine aminotransferase (ALT), aspartate aminotransferase (AST) and glutathione peroxidase activity (GSH‐Px)

The alanine aminotransferase (ALT) assay Kit (C009‐2, NanJing JianCheng Bioengineering Institute, Nanjing, China), aspartate aminotransferase (AST) assay Kit (C010‐2, NanJing JianCheng Bioengineering Institute, Nanjing, China) and glutathione peroxidase activity (GSH‐Px) assay Kit (A005, NanJing JianCheng Bioengineering Institute, Nanjing, China) were used for measuring the level of serum ALT, AST and liver GSH‐Px according to the instructions of the manufacturer.

### 2.5 Western blot analysis

The protein extraction and Western blot analysis were performed as previous described.^22^ The antibody information was as following: kindlin‐2 antibody (13562s, Cell Signaling Technology), Bax antibody (14796, Cell Signaling Technology), Bcl‐2 antibody (ab194583, Abcam), GRP78 antibody (GRP78, 3183, Cell Signaling Technology), CHOP antibody (2895, Cell Signaling Technology), PDI antibody (3501, Cell Signaling Technology), Ero1‐Lα antibody (ab81959, Abcam), Drp‐1 antibody (ab184247, Abcam), Tfam antibody (ab131607, Abcam), ND3 (ab192306, Abcam), Mfn‐2 (9482, Cell Signaling Technology), Fis‐1 (ab71498, Abcam), ATPB (ab170947, Abcam), β actin Antibody (HRP‐60008, Proteintech), Goat anti‐rabbit IgG antibody (SA00001‐2, Proteintech) and Goat anti‐mouse IgG antibody (SA00001‐1, Proteintech). The Image J software was used for quantitative analysing, and relative protein levels were expressed as the intensity ratio of target protein and β actin.

### 2.6 RNA extraction, reverse transcription and quantitative PCR (q‐PCR)

RNA was extracted from liver samples using TRIzol Reagent (9108, TAKARA BIO INC). One thousand nanograms of RNA was reverse transcribed to cDNA using PrimeScript™ RT Master Mix (RR036A, TAKARA BIO INC) and amplified by q‐PCR using SYBR green PCR Master Mix (RR820A, TAKARA BIO INC). The following primer sets (TAKARA BIO INC) were used: mouse IL‐1β (forward 5′ TCC AGG ATG AGG ACA TGA GCA C 3′; reverse, 5′ GAA CGT CAC ACA CCA GCA GGT TA 3′), mouse IL‐6 (forward 5′ CCA CTT CAC AAG TCG GAG GCT TA 3′; reverse, 5′ TGC AAG TGC ATC ATC GTT GTT C 3′), mouse MCP‐1 (forward 5′ AGC AGC AGG TGT CCC AAA GA 3′; reverse, 5′ GTG CTG AAG ACC TTA GGG CAG A 3′), mouse CXCL‐1 (forward 5′ TGC ACC CAA ACC GAA GTC 3′; reverse, 5′ GTC AGA AGC CAG CGT TCA CC 3′), mouse Fis‐1 (forward 5′ TGG GCA ACT ACC GGC TCA A 3′; reverse, 5′ TTA TCA ATC AGG CGT TCC AGC TC 3′), mouse PGC‐1α (forward 5′ AAC AGG AAC AGC AGC AGA GAC A 3′; reverse, 5′ GAG GAG TTG TGG GAG GAG TTA GG 3′) and mouse β actin (endogenous control; forward 5′ GTG ACG TTG ACA TCC GTA AAG A 3′; reverse, 5′ GTA ACA GTC CGC CTA GAA GCA C 3′).

### 2.7 Immunohistochemistry and immunofluorescence staining

Immunohistochemistry and immunofluorescence staining were performed as previous described.^17^ The MPO antibody (Santa Cruz Biotechnology, Inc) was used for detection of liver MPO expression by immunohistochemistry staining. Liver Terminal Deoxynucleotidyl Transferase‐Mediated dUTP Nick End Labelling Assay (TUNEL, Roche), CD11b antibody (GB11058, Servicebio) and Dihydroethidium (DHE) dye (D7008, Sigma‐aldrich) were used for detection of liver apoptosis cells, CD11b positive inflammatory cells and reactive oxygen species (ROS) levels via immunofluorescence staining. Image J software was used to quantitative analysis.

### 2.8 Cell culture, lipotoxic induction and hypoxia/reoxygenation (H/R)

HL‐7702 cells (Normal human hepatocytes) were cultured in RPMI‐1640 medium with 10% foetal bovine serum (FBS) and 100 units/ml penicillin/ streptomycin mixture (Gibco) at 37°C with 100% humidity and 5% CO_2_ in vitro. HL‐7702 cells were treated with palmitic acid (PA, 0.2 mmol/L) and oleic acid (OA, 0.1 mmol/L) to induce lipotoxicity injury of hepatocytes. Cells were deprived of oxygen (94% N_2_, 5% CO_2_, 1% O_2_) in a serum‐free and deoxyglucose‐rich (5 mmol/L) medium to simulate hepatic ischaemia and hypoxia of mice for 1 hour; then, the cells were changed to 5% CO_2_ condition with RPMI‐1640 medium containing 10% FBS for 6 hours.

### 2.9 Transfection of small interfering RNA (siRNA)

The siRNA was constructed by GenePharma Corporation and transfected according to the experimental instructions. The siRNA‐kindlin‐2:5′‐GCU UCC CAA CAU GAA GUA UTT‐3′ and 5′‐AUA CUU CAU GUU GGG AAG CTT‐3′ (GenePharma) was used to deplete the expression of kindlin‐2 cells and a siRNA‐negative control: 5′‐UUC UCC GAA CGU GUC ACG UTT‐3′ and 5′‐ACG UGA CAC GUU CGG AGA ATT‐3′ (GenePharma) was used as negative control in HL‐7702 cells.

### 2.10 Statistical analysis

The data were expressed as mean ± standard error (SE). The *t* test or one‐way ANOVA was used to analyse the differences between groups. SPSS version 18.0 (IBM) was used for statistical analysis, and *P* value < .05 was considered statistically significant.

## 3 RESULTS

### 3.1 HFD exaggerates I/R‐induced liver injury

**Figure 1:**
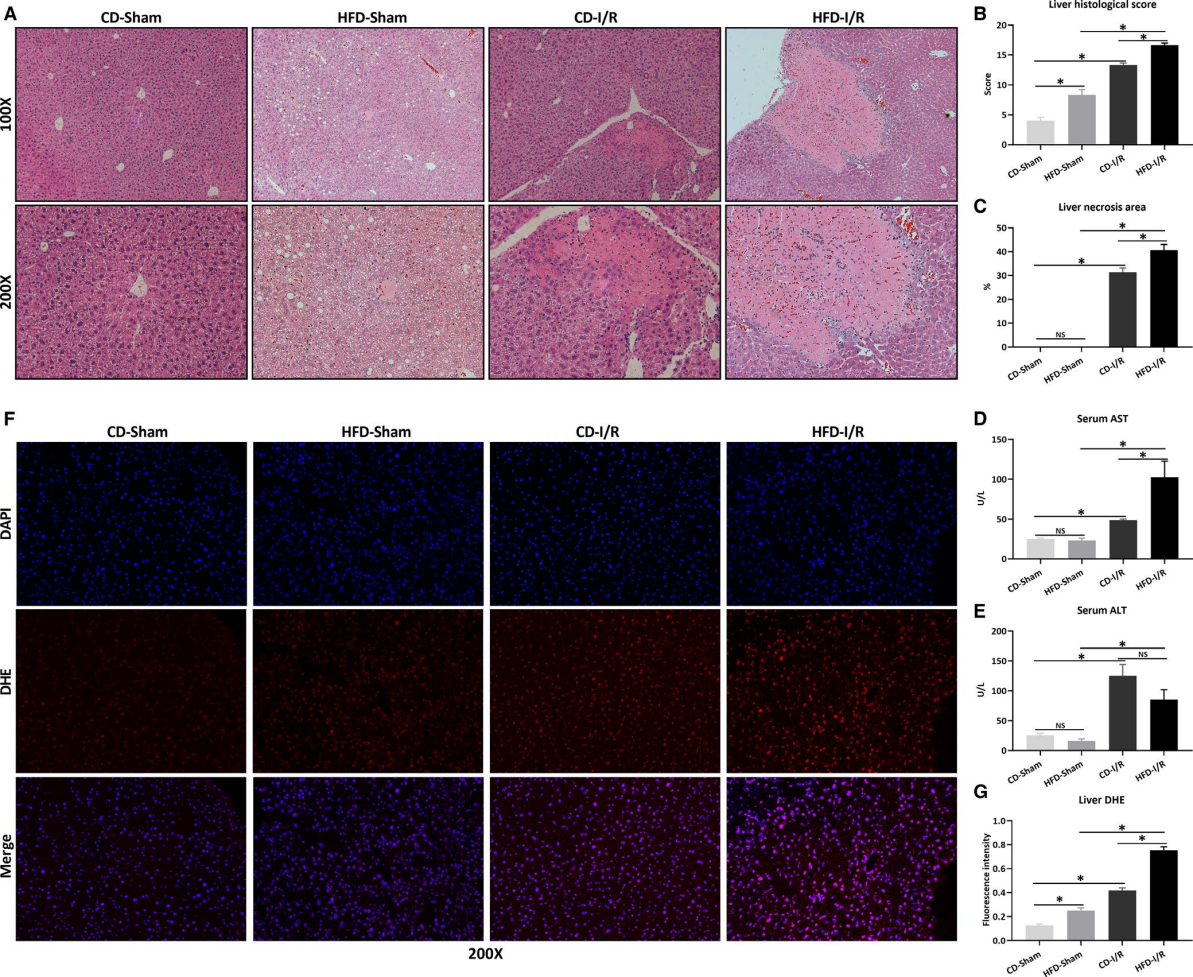
HFD exaggerates I/R‐induced liver injury. Hepatic ischaemia was induced by occluding partial (70%) hepatic arterial/portal venous blood for 60 min, followed by 24 h of reperfusion. Sham mice underwent all the procedures except hepatic ischaemia. After 12 wk of control diet (CD) or high‐fat diet (HFD), mice underwent sham operation (CD‐Sham, HFD‐Sham) or hepatic I/R (CD‐I/R, HFD‐I/R). Liver H&E staining (A), histological score (B) and necrosis area (C). Original magnification, 100× and 200×. The levels of serum AST (D) and ALT (E). F, Liver DHE staining (red) and counterstained with DAPI (blue). Original magnification, 200×. G, The quantitative analysis of liver DHE fluorescence intensity. Results are expressed as mean ± SE (n = 4‐5/group) and compared by t test or one‐way ANOVA. *P < .05, ^NS^ *P* > .05

To study the effects of HFD on hepatic I/R injury, mice were fed either a control diet (CD) or a high‐fat diet (HFD) for 12 weeks. As showed in Figure S1A, HFD‐fed mice were heavier than CD‐fed mice (*P* < .05). HFD‐fed mice were associated with more prominent liver histological damage and more fat content (Figures S1B‐D, *P* < .05). Then, HFD‐fed and CD‐fed mice underwent hepatic I/R (CD‐I/R or HFD‐I/R) or sham operation (CD‐Sham or HFD‐Sham). Figure S2A showed that hepatic I/R increased serum irisin level in HFD‐fed mice (*P* < .05). As showed in Figure 1A,B, HFD induced significant liver injury characterized by hepatic steatosis (*P* < .05). After I/R, HFD‐fed mice showed more severe liver injury and larger necrosis area than CD‐fed mice (Figure 1A‐C, *P* < .05). The changes of serum AST and ALT were consistent with histological lesions (Figure 1D,E, *P* < .05). In addition, HFD exaggerated I/R‐induced ROS production (Figure 1F,G, *P *< .05).

### 3.2 Irisin attenuates hepatic I/R injury in HFD‐fed mice

**Figure 2:**
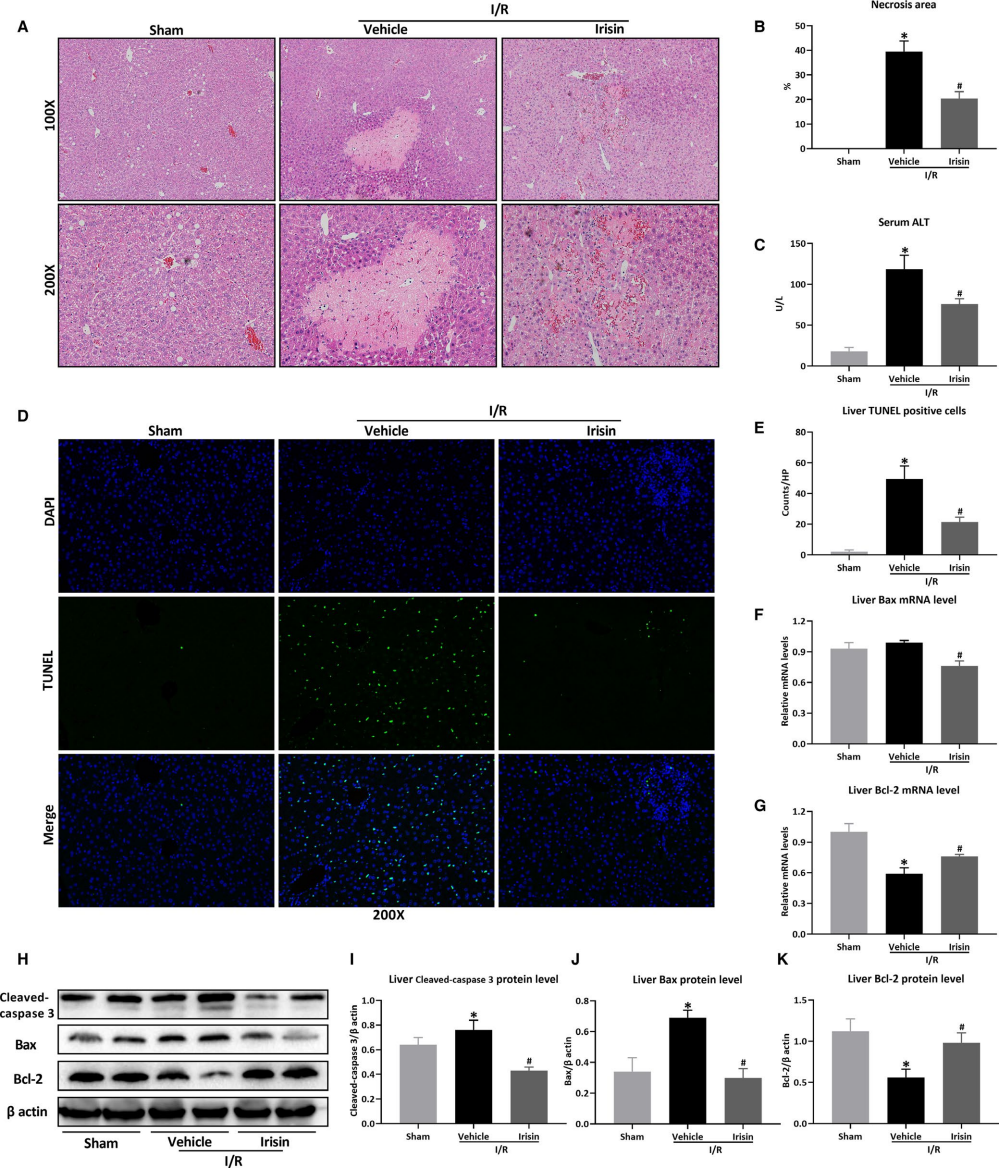
Irisin attenuates hepatic I/R injury in HFD‐fed mice. Hepatic ischaemia was induced by occluding partial (70%) hepatic arterial/portal venous blood for 60 min, followed by 24 h of reperfusion. Sham mice underwent all the procedures except hepatic ischaemia. After 12 wk of high‐fat diet (HFD), HFD‐fed mice underwent sham operation (Sham) or hepatic I/R treated with 0.5 mL saline (Vehicle) or irisin (250 μg/kg, 0.5 mL). Liver H&E staining (A) and necrosis area (B). Original magnification, 100× and 200×. C, The level of serum ALT. D, Liver TUNEL staining (green) and counterstained with DAPI (blue). Original magnification, 200×. E, The quantitative analysis of liver TUNEL positive cells. Liver relative mRNA levels of Bax (F) and Bcl‐2 (G). Western blot analysis of Bax and Bcl‐2 (H) and the quantitative analysis of Bax (I) and Bcl‐2 (J). Results are expressed as mean ± SE (n = 4‐5/group) and compared by *t* test or one‐way ANOVA. *P < .05 vs sham group, ^#^ *P* < .05 vs vehicle group

Next, the effect of irisin on hepatic I/R injury in HFD‐fed mice was explored. Figure 2A,B demonstrated that irisin administration reduced I/R‐induced hepatic necrosis in HFD‐fed mice (*P* < .05). Similarly, serum ALT levels were also significantly decreased by irisin treatment (118.6 ± 17.0 U/L vs 76.1 ± 6.2 U/L, Figure 2C, *P *< .05). Figure 2D,E indicated that TUNEL positive cells increased markedly at 24 hours after I/R in HFD‐fed mice. Irisin treatment significantly decreased liver TUNEL positive cells after I/R in HFD‐fed mice (*P* < .05). Consistently, irisin treatment reduced the expression of cleaved‐caspase 3 at the protein level (Figure 2H,I) and decreased the expression of Bax in mRNA (Figure 2F) and protein levels (Figure 2H,J), while increased the expression of Bcl‐2 in mRNA (Figure 2G) and protein levels (Figure 2H,K) after hepatic I/R in HFD‐fed mice (all *P* < .05).

### 3.3 Irisin alleviates oxidative stress after hepatic I/R in HFD‐fed mice

**Figure 3:**
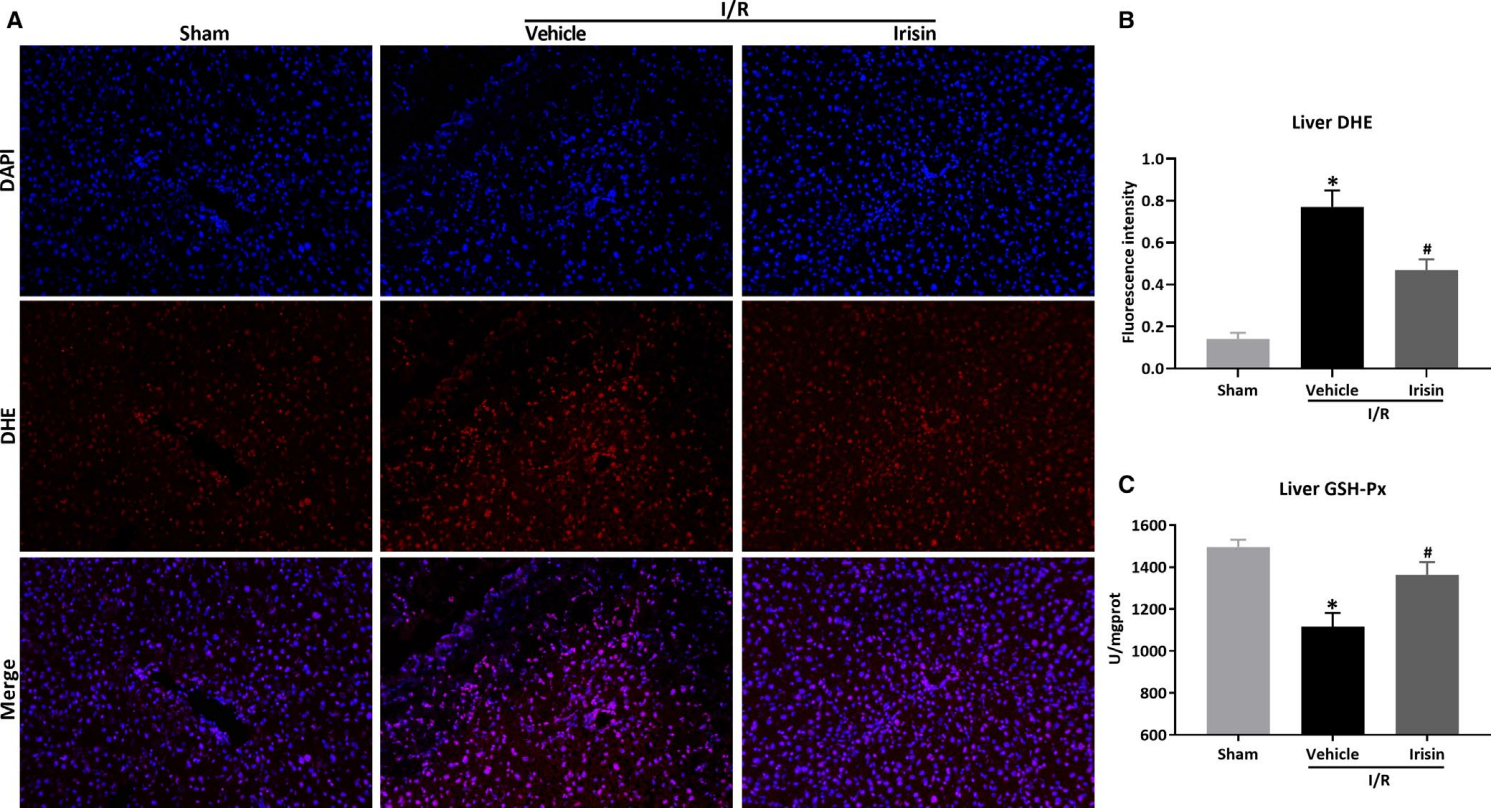
Irisin alleviates oxidative stress after hepatic I/R in HFD‐fed mice. Hepatic ischaemia was induced by occluding partial (70%) hepatic arterial/portal venous blood for 60 min, followed by 24 h of reperfusion. Sham mice underwent all the procedures except hepatic ischaemia. After 12 wk of high‐fat diet (HFD), HFD‐fed mice underwent sham operation (Sham) or hepatic I/R treated with 0.5 mL saline (Vehicle) or irisin (250 μg/kg, 0.5 mL). A, Liver DHE staining (red) and counterstained with DAPI (blue). Original magnification, 200×. B, The quantitative analysis of liver DHE fluorescence intensity. C, The level of liver GSH‐Px. Results are expressed as mean ± SE (n = 4‐5/group) and compared by *t* test or one‐way ANOVA. *P < .05 vs sham group, ^#^ *P* < .05 vs vehicle group

Then, the effects of irisin on oxidative stress were evaluated. As shown by liver DHE staining in Figure 3A,B, irisin administration inhibited the production of ROS compared to vehicle‐treated mice (*P* < .05). Antioxidant GSH‐Px decreased in hepatic I/R and increased after irisin administration in HFD‐fed mice (Figure 3C, *P *< .05).

### 3.4 Irisin improves mitochondrial function and reduces ER stress after hepatic I/R in HFD‐fed mice

**Figure 4:**
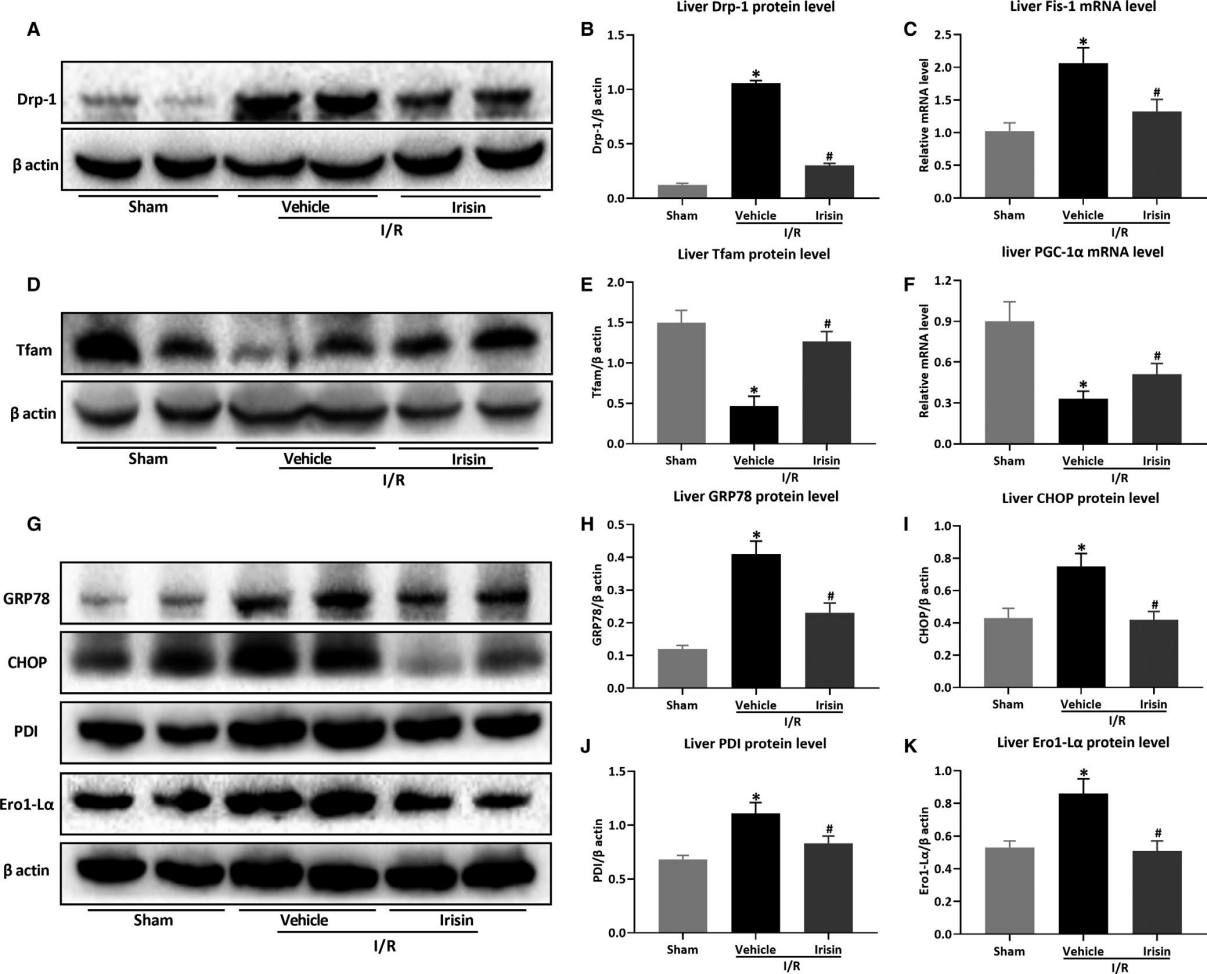
Irisin improves mitochondrial function and reduces ER stress after hepatic I/R in HFD‐fed mice. Hepatic ischaemia was induced by occluding partial (70%) hepatic arterial/portal venous blood for 60 min, followed by 24 h of reperfusion. Sham mice underwent all the procedures except hepatic ischaemia. After 12 wk of high‐fat diet (HFD), HFD‐fed mice underwent sham operation (Sham) or hepatic I/R treated with 0.5 mL saline (Vehicle) or irisin (250 μg/kg, 0.5 mL). Western blot analysis of Drp‐1 (A) and its quantitative analysis (B). C, Liver relative mRNA level of Fis‐1. Western blot analysis of Tfam (D) and its quantitative analysis (E). F, Liver relative mRNA level of PGC‐1α. Western blot analysis of ER stress‐related proteins (G) and their quantitative analysis of liver GRP78 (H), CHOP (I), PDI (J) and Ero1‐Lα (K). Results are expressed as mean ± SE (n = 4‐5/group) and compared by *t* test or one‐way ANOVA. *P < .05 vs sham group, ^#^ *P* < .05 vs vehicle group

Figure 4A‐C showed that irisin inhibited the excessive expression of mitochondrial fission proteins Drp‐1 and Fis‐1 at mRNA or protein levels (*P* < .05). Moreover, irisin treatment also restored the reduced expression of mitochondrial biogenesis proteins PGC‐1α and Tfam (Figure 4D‐F, *P *< .05). As showed in Figure 4G‐K, the expressions of liver endoplasmic reticulum (ER) stress‐related proteins (GRP78, CHOP, PDI and Ero1‐Lα) were significantly up‐regulated after hepatic I/R in HFD‐fed mice but reduced after irisin administration (*P* < .05).

### 3.5 Irisin inhibits inflammatory response after hepatic I/R in HFD‐fed mice

**Figure 5:**
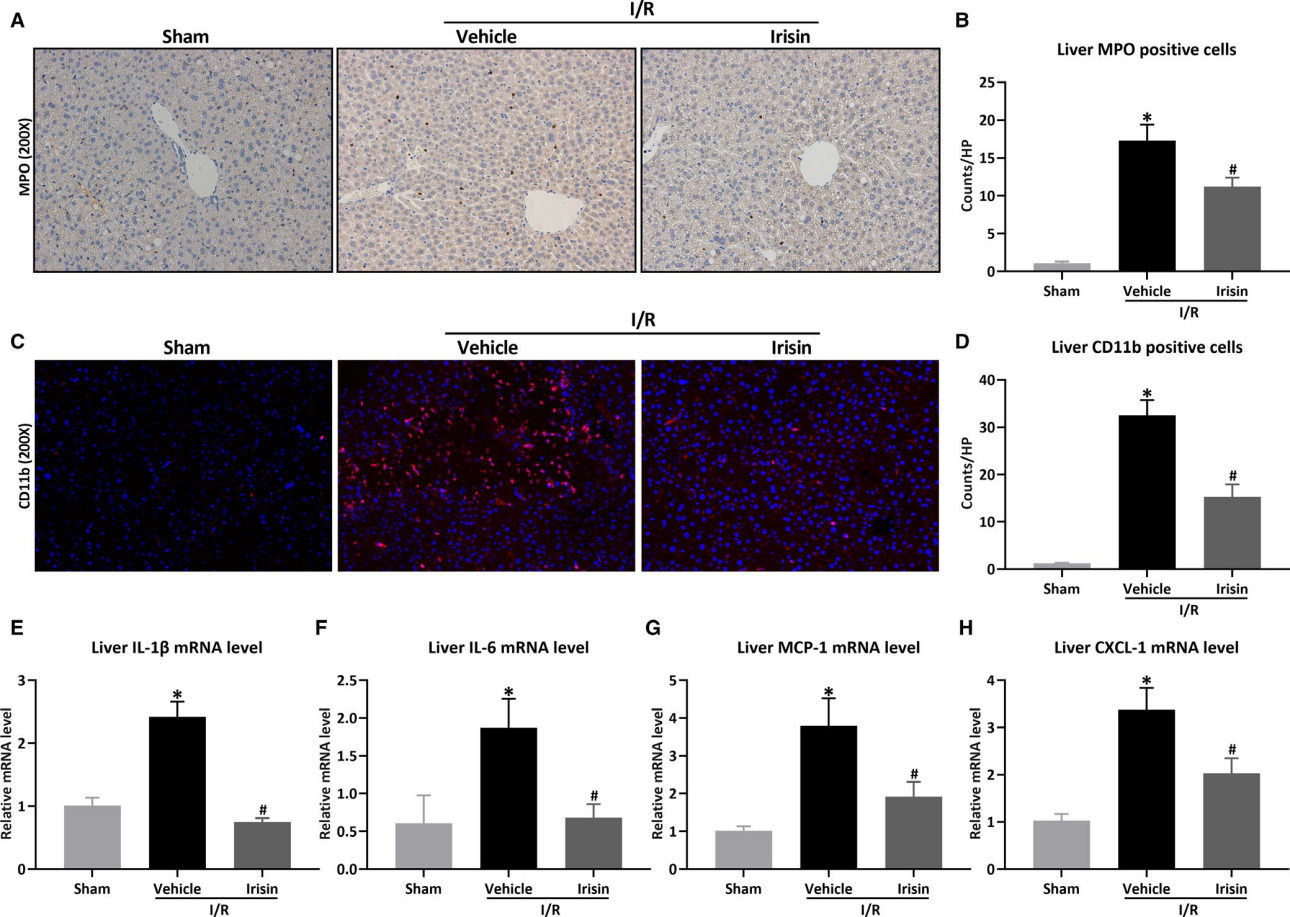
Irisin inhibits inflammatory response after hepatic I/R in HFD‐fed mice. Hepatic ischaemia was induced by occluding partial (70%) hepatic arterial/portal venous blood for 60 min, followed by 24 h of reperfusion. Sham mice underwent all the procedures except hepatic ischaemia. After 12 wk of high‐fat diet (HFD), HFD‐fed mice underwent sham operation (Sham) or hepatic I/R treated with 0.5 mL saline (Vehicle) or irisin (250 μg/kg, 0.5 mL). Liver MPO staining (A) and its quantitative analysis of liver MPO positive cells (B). Original magnification, 200×. Liver CD11b staining (red) (C) and its quantitative analysis of liver CD11b positive cells (D). Original magnification, 200×. Liver relative mRNA levels of IL‐1β (E), IL‐6 (F), MCP‐1 (G), CXCL‐1 (H). Results are expressed as mean ± SE (n = 4‐5/group) and compared by *t* test or one‐way ANOVA. *P < .05 vs sham group, ^#^ *P* < .05 vs vehicle group

Inflammatory response was evaluated after hepatic I/R in HFD‐fed mice. The recruitment of neutrophils and macrophages was detected by liver MPO and CD11b immunostaining, while the release of inflammatory factors was detected by q‐PCR. In Figure 5A‐D, hepatic I/R increased liver MPO and CD11b positive cells in HFD‐fed mice, which were reversed after irisin administration (*P* < .05). Consistently, compared to HFD‐fed mice of hepatic I/R, the mRNA levels of liver inflammatory factors (IL‐1β, IL‐6, MCP‐1 and CXCL‐1) were also reduced after irisin administration in HFD‐fed mice (Figure 5E‐H, *P* < .05).

### 3.6 Kindlin‐2 inhibition by RNAi eliminates irisin's direct effects on cultured hepatocytes

**Figure 6:**
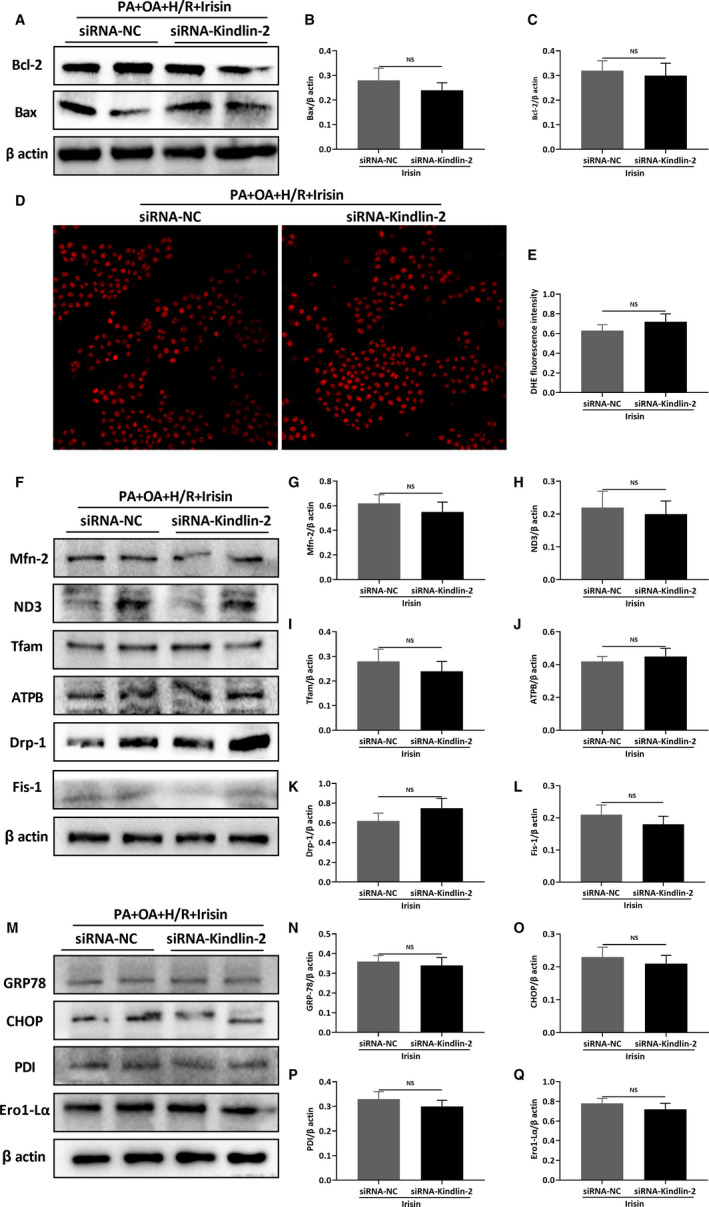
The knockdown of kindlin‐2 eliminates the protective effect of irisin. The HL‐7702 cells were transfected with siRNA of kindlin‐2 (or negative control) for 48 h and treated with palmitic acid (PA, 0.2 mmol/L) and oleic acid (OA, 0.1 mmol/L) to induce lipotoxicity injury of hepatocytes. Cells were deprived of oxygen (94% N_2_, 5% CO_2_, 1% O_2_) in a serum‐free and deoxyglucose‐rich (5 mmol/L) medium to simulate hepatic ischaemia and hypoxia of mice for 1 h; then, the cells were changed to 5% CO_2_ condition with RPMI‐1640 medium containing 10% FBS for 6 h. Western blot analysis of Bax and Bcl‐2 (A) and their quantitative analysis of Bax (B) and Bcl‐2 (C). DHE staining (red) (D) and its quantitative analysis of DHE fluorescence intensity (E). Western blot analysis of mitochondrial related proteins (F) and their quantitative analysis of Mfn‐2 (G), ND3 (H), Tfam (I), ATPB (J), Drp‐1 (K) and Fis‐1 (L). Western blot analysis of ER stress‐related proteins (M) and their quantitative analysis of GRP78 (N), CHOP (O), PDI (P) and Ero1‐Lα (Q). Results are expressed as mean ± SE (n = 3‐4/group) and compared by *t* test. ^NS^ *P* > .05

To investigate the role of kindlin‐2 in irisin's biological function, we knocked down kindlin‐2 expression in HL‐7702 cells (Figure S3A,B, *P* < .05). The cells were exposed to palmitic acid (PA, 0.2 mmol/L) and oleic acid (OA, 0.1 mmol/L) to induce lipotoxicity injury and then subjected to hypoxia and reoxygenation treatment with/without the presence of irisin. As shown in Figure 6, kindlin‐2 knockdown eliminated the effects of irisin on cell apoptosis (Figure 6A‐C), ROS production (Figure 6D,E), mitochondrial function (Figure 6F‐L) and ER stress (Figure 6M‐Q) (all *P* < .05). Meanwhile, the effect of irisin on kindlin‐2 was detected after irisin treatment in HFD‐fed mice and irisin did not change the expression of kindlin‐2 (Figure S4A,B, *P* > .05).

## 4 DISCUSSIONS

**Figure 7:**
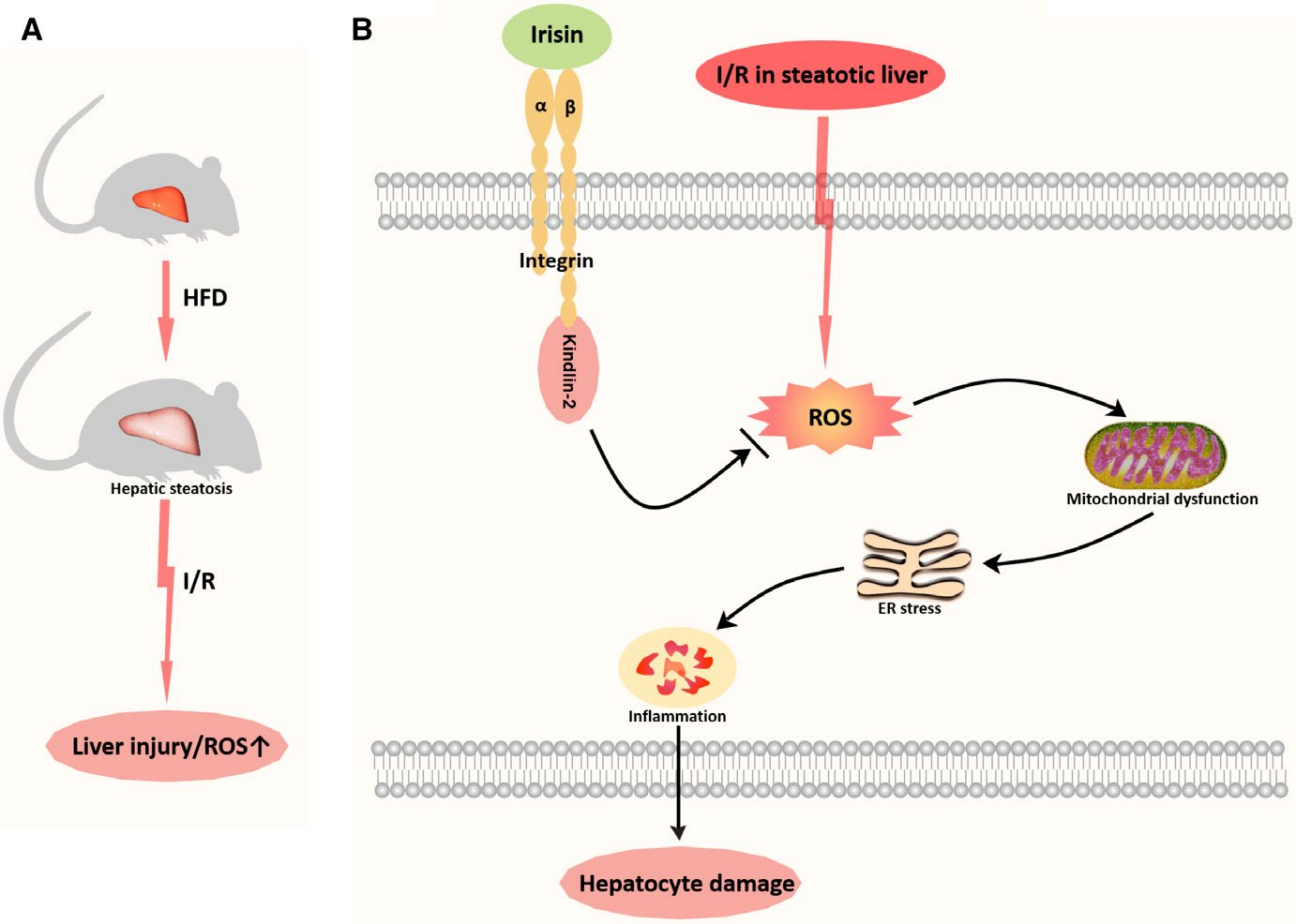
Irisin attenuates hepatic I/R injury via a kindlin‐2 dependent mechanism in steatotic liver. HFD exaggerated I/R‐induced liver injury and ROS production. And irisin administration attenuated liver injury, improved mitochondrial function, and reduced oxidative and ER stress in HFD‐fed hepatic I/R mice. However, inhibition of kindlin‐2 by RNAi eliminated irisin's effects on apoptosis, mitochondrial function, oxidative and ER stress in cultured hepatocytes

In the present study, using an established model of hepatic I/R in HFD‐fed mice, we found that HFD exaggerated I/R‐induced liver injury and ROS production. And irisin administration attenuated hepatic injury, improved mitochondrial function, and reduced oxidative and ER stress in HFD‐fed hepatic I/R mice. However, in cultured hepatocytes, inhibition of kindlin‐2 by RNAi eliminated irisin's effects on apoptosis, mitochondrial function, oxidative and ER stress (Figure 7).

Hepatic I/R is a life‐threaten complication in liver surgery and associated with significant morbidity and mortality, especially in hepatic I/R of steatotic liver.^6^, ^23^, ^24^, ^25^, ^26^, ^27^ Research has shown that about 20% of patients undergoing hepatectomy have various degrees of hepatic steatosis.^28^ Moreover, steatotic liver (about 20%‐30% of liver donors) has been introduced as the most common type of ‘extended criteria’ organs due to organ shortage.^29^, ^30^, ^31^ However, hepatic steatosis exaggerates I/R‐induced liver injury, which has been proven in clinical and experimental studies.^6^, ^23^, ^24^, ^25^, ^26^, ^27^ Although the exact mechanism remains unclear, ROS is believed to play an important role in both hepatic steatosis^32^, ^33^ and I/R injury.^34^, ^35^, ^36^ In NAFLD, fat‐laden hepatocytes are damaged by chronic oxidative/nitrosative stress (ONS). And ONS is acutely exacerbated during hepatic I/R, leading to extensive parenchymal damage.^7^ In the present study, we also showed more severe liver injury and ROS production after hepatic I/R in HFD‐fed mice, suggesting inhibition of ROS production may be an effective therapeutic target.

Irisin, an exercise‐induced hormone, has emerged as a key regular of energy homeostasis in obesity, diabetes and NAFLD.^37^, ^38^, ^39^, ^40^ A recent study has shown that irisin expression increased in non‐parenchymal cells of fatty liver and was associated with the increase in innate immune cells (ie CD11b positive cells).^41^ In the present study, liver CD11b positive cells were increased significantly in HFD‐hepatic I/R mice, which may be the reason for the increase of serum irisin levels under such conditions. We and others have shown irisin plays a protective role in I/R of multiple organs and improvement of mitochondrial function and oxidative stress are the most common mechanisms.^9^, ^10^, ^11^, ^12^, ^13^, ^14^, ^15^, ^16^, ^17^ However, the effect of irisin on I/R in steatotic liver remained unknown. The present study is the first one to reveal that irisin attenuated liver injury, improved mitochondrial function, and reduced oxidative and ER stress after I/R in steatotic liver. The dosage of irisin was based on our previous study.^17^ As shown in the present study, it was also protective in HFD‐hepatic I/R mice. However, the optimal dosage and the dose‐dependent effect of irisin in HFD‐hepatic I/R warrants further investigation. Hepatic I/R is accompanied by increased production of ROS. Mitochondria are a main source of ROS and ROS impairs mitochondrial function.^42^, ^43^, ^44^, ^45^ Endoplasmic reticulum (ER) stress is closely related to mitochondrial dysfunction. ER stress inhibition protects steatotic and non‐steatotic liver from hepatic I/R.^46^, ^47^, ^48^ ER‐stressed steatotic hepatocytes activate apoptotic and inflammatory pathways in hepatic I/R and NAFLD,^49^, ^50^ leading to liver injury.

Kindlin‐2, a member of kindlins, directly interacts with the cytoplasmic tail of β integrin to mediate cell adhesion, cell motility, cytoskeletal organization, cell survival, gene transcription and cell proliferation.^51^, ^52^, ^53^ Kindlin‐2 has been shown to promote tumour invasion and metastasis.^54^, ^55^ It is also essential for preserving integrity of the heart, vascular permeability in angiogenesis, chondrogenesis, regulation of podocyte structure and function, control of adipogenesis and lipid metabolism as well as bone homeostasis.^56^, ^57^, ^58^, ^59^, ^60^, ^61^ Kindlins protect cells against oxidative damage.^62^, ^63^, ^64^, ^65^ Ling Guo et al have discovered that depletion of kindlin‐2 increased ROS production.^66^ αVβ5 integrin was reported to be the receptor of irisin,^21^ and our recent studies have shown irisin mitigated I/R injury via binding to αVβ5 integrin.^18^ However, the effect of irisin on kindlin‐2, an important regulator of αVβ5 integrin function, remained unknown. In the present study, we found irisin did not change the expression of kindlin‐2 after hepatic I/R in HFD‐fed mice. However, kindlin‐2 knockdown by RNAi eliminated the beneficial effects of irisin in hypoxia/reoxygenation‐treated hepatocytes, suggesting kindlin‐2 is involved in irisin's biological function. However, whether depletion of Kindlin‐2 inhibits irisin induced protection in hepatic IR in the HFD‐mice warrants further investigation. And the detailed mechanism of kindlin‐2 after hepatic I/R in the HFD‐mice will be further explored in our future studies.

In summary, using a model of hepatic I/R in HFD‐fed mice, we demonstrated that irisin attenuates I/R injury in steatotic liver. The protective effect of irisin under such conditions requires kindlin‐2. Irisin may be a novel effective treatment for NAFLD patients with hepatic I/R.

## CONFLICT OF INTEREST

The authors have no conflicts of interest to disclose.

## AUTHOR CONTRIBUTIONS

**Jia Zhang:** Conceptualization (equal); Data curation (lead); Formal analysis (equal); Methodology (equal); Project administration (equal). **Yifan Ren:** Data curation (equal). **Jianbin Bi:** Data curation (equal). **Mengzhou Wang:** Data curation (equal). **Lin Zhang:** Data curation (equal). **Tao Wang:** Data curation (equal). **Shasha Wei:** Data curation (equal); Funding acquisition (equal). **Xingyi Mou:** Data curation (equal). **Yi Lv:** Conceptualization (supporting); Project administration (supporting). **Rongqian Wu:** Conceptualization (lead); Funding acquisition (lead); Project administration (lead).

## Supporting information

Fig S1‐S4

## Data Availability

The data that support the findings of this study are available from the corresponding author upon reasonable request.
