# Supplementary material for: Involvement of kindlin‐2 in irisin’s protection against ischaemia reperfusion‐induced liver injury in high‐fat diet‐fed mice
Source: J Cell Mol Med. 2020 Sep 20;24(22):13081–92. doi: 10.1111/jcmm.15910 (PMC7701503; doi:10.1111/jcmm.15910)
Supplement: Supplementary file 1 — Fig S1‐S4 [file JCMM-24-13081-s001.docx]

**Involvement of Kindlin-2 in Irisin’s Protection Against Ischemia Reperfusion-Induced Liver Injury in High Fat Diet-Fed Mice**

**Supplemental Materials:**


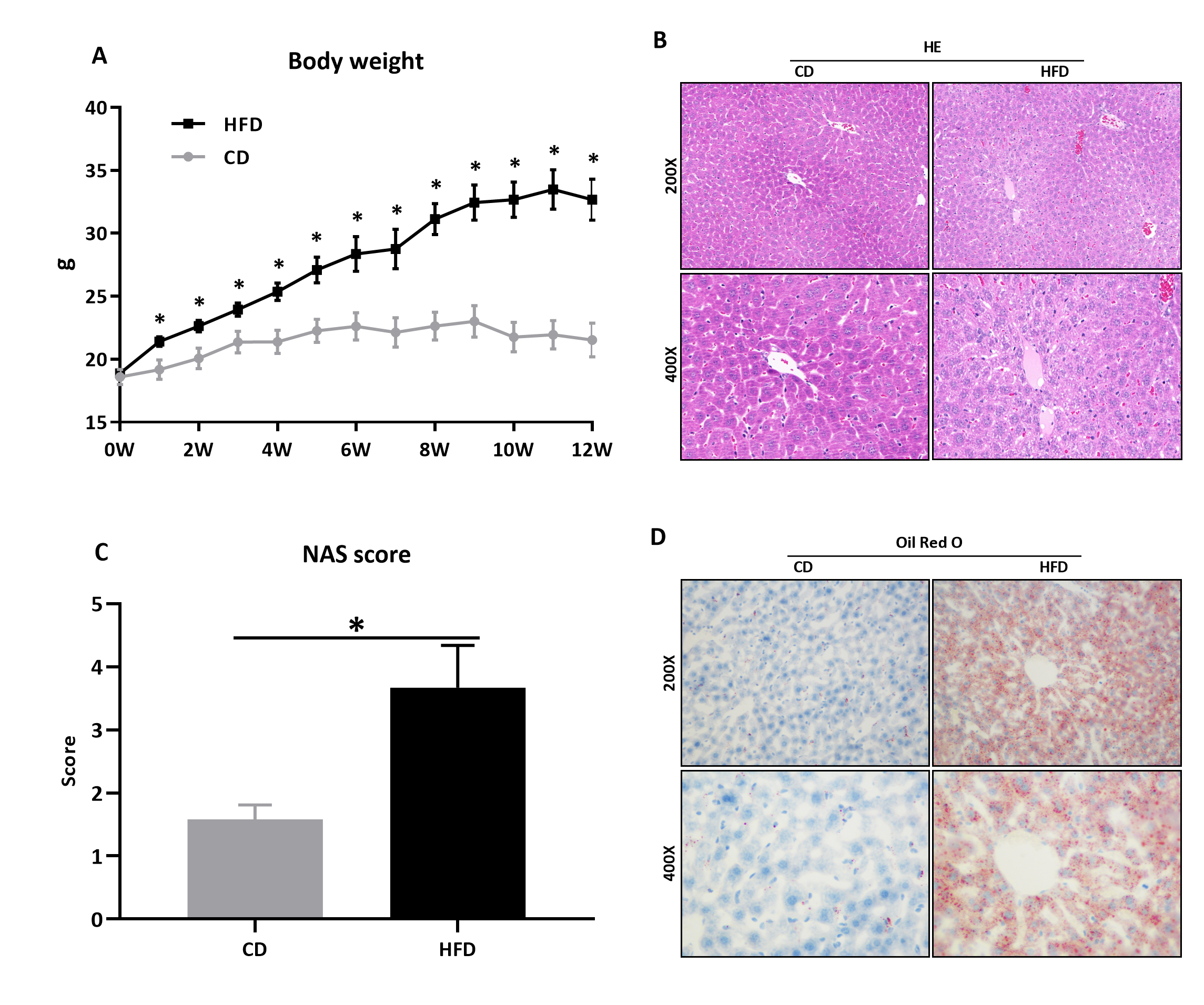


**Figure S1. The mice on the high-fat diet had increased body weight and hepatic steatosis. A,** The body weight of CD- and HFD-fed mice. Liver H&E staining **(B)** and NAS score **(C)** of CD- and HFD-fed mice. Original magnification, x200 and x400. **D,** Liver Oil Red O staining of CD- and HFD-fed mice. Original magnification, x200 and x400. Results are expressed as mean ± SE (n=4-5/group) and compared by t-test. * p < 0.05.


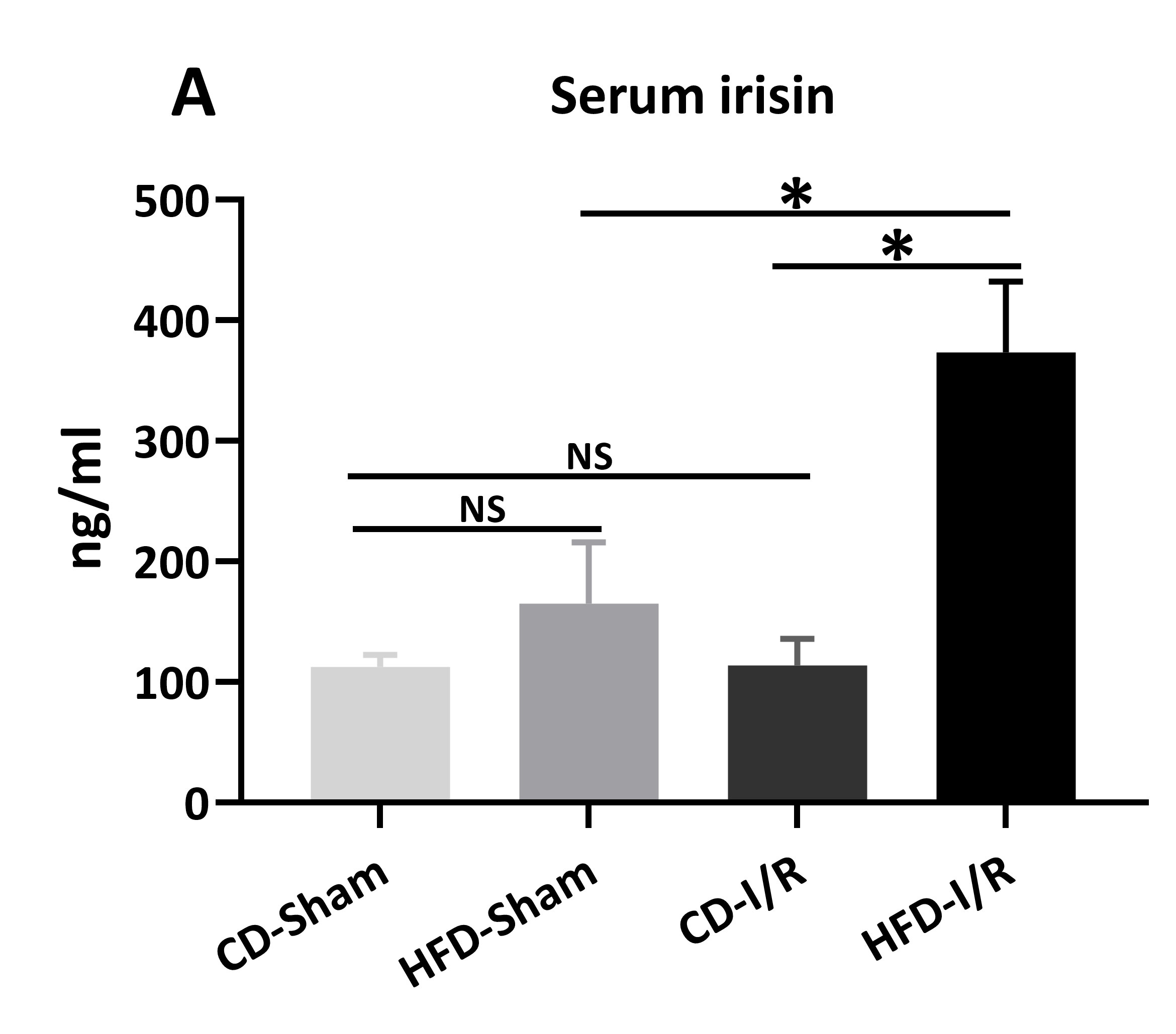


**Figure S2. Hepatic I/R increased serum irisin level in HFD-fed mice.** **A,** the level of serum irisin in CD- and HFD-fed mice after hepatic I/R. ^NS^ p > 0.05, * p < 0.05.


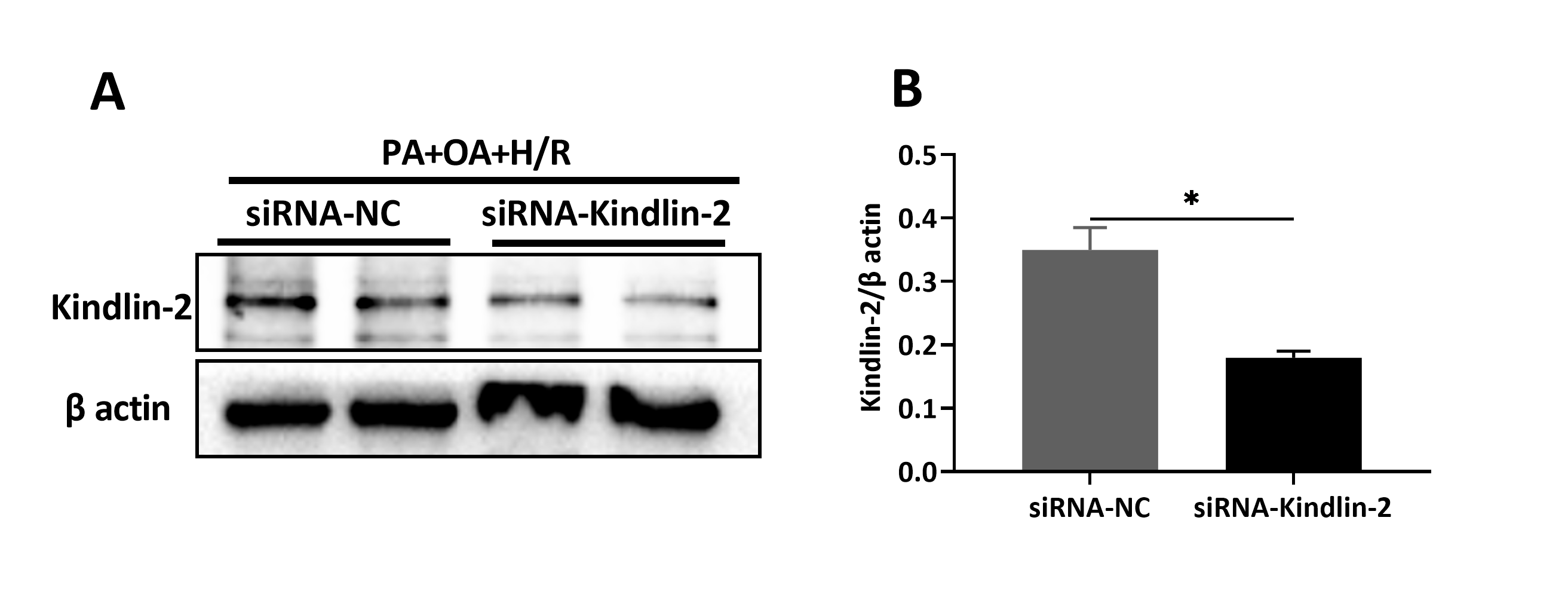


**Figure S3. The expression of kindlin-2 was knocked down by siRNA-Kindlin-2.** Western blot analysis of kindlin-2 **(A)** and its quantitative analysis **(B)** in cultured hepatocytes. * p < 0.05.


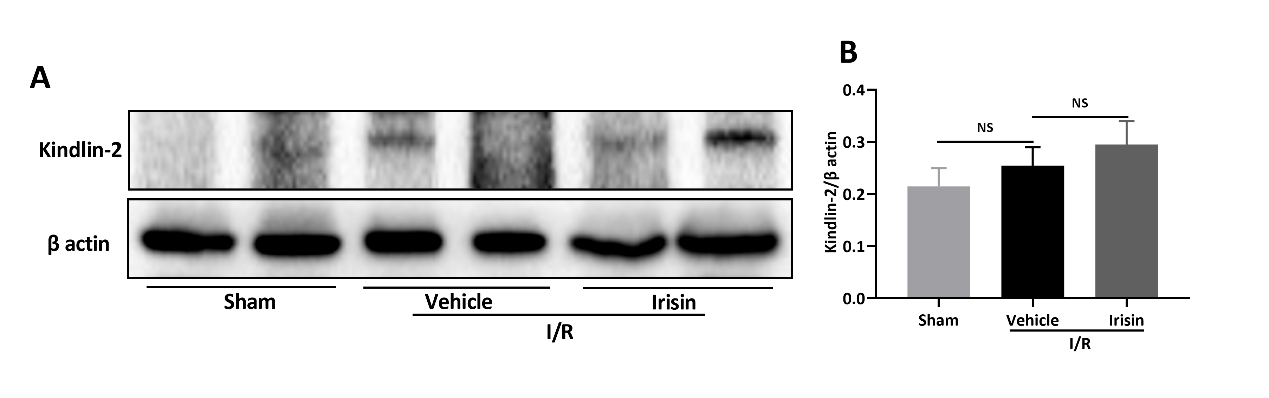


**Figure S4. Irisin did not change the expression of kindlin-2.** Western blot analysis of kindlin-2 **(A)** and its quantitative analysis **(B)** after hepatic I/R in HFD-fed mice. ^NS^ p > 0.05.
